# Inhibition of Bruton’s Tyrosine Kinase Modulates Microglial Phagocytosis: Therapeutic Implications for Alzheimer’s Disease

**DOI:** 10.1007/s11481-019-09839-0

**Published:** 2019-02-13

**Authors:** James Keaney, Julien Gasser, Gaëlle Gillet, Diana Scholz, Irena Kadiu

**Affiliations:** Neuroscience Therapeutic Area, New Medicines, UCB Biopharma SPRL, Chemin du Foriest, 1420 Braine-l’Alleud, Belgium

**Keywords:** Neuroinflammation, Microglia, Bruton’s tyrosine kinase, Alzheimer’s disease, Phagocytosis, Phospholipase gamma 2

## Abstract

Bruton’s tyrosine kinase (BTK), a critical component of B cell receptor signaling, has recently been implicated in regulation of the peripheral innate immune response. However, the role of BTK in microglia, the resident innate immune cells of the central nervous system, and its involvement in the pathobiology of neurodegenerative disease has not been explored. Here we found that BTK is a key regulator of microglial phagocytosis. Using potent BTK inhibitors and small interfering RNA (siRNA) against BTK, we observed that blockade of BTK activity decreased activation of phospholipase gamma 2, a recently identified genetic risk factor in Alzheimer’s disease (AD), and reduced phagocytosis in rodent microglia and human monocyte-derived macrophages. Inhibition of BTK signaling also decreased microglial uptake of synaptosomes but did not have major impacts on other key microglial functions such as migration and cytokine release. Similarly, blocking BTK function ex vivo in acute brain slices reduced microglial phagocytosis and maintained numbers of resting microglia. In brain tissues from the 5xFAD mouse model of AD, levels of microglial BTK were elevated while in two gene expression datasets of post-mortem AD patient brain tissues, upregulation of BTK transcript was observed. Our study provides novel insights into the role of BTK in regulating microglial phagocytosis and uptake of synaptic structures and suggests that inhibiting microglial BTK may improve cognition in AD by preventing microglial activation and synaptic loss.

**Graphical Abstract:**
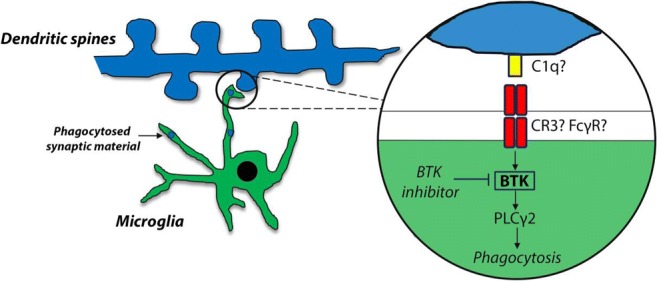
Microglial-mediated synapse loss has been implicated in AD pathogenesis. Inhibition of BTK decreases activation of PLCγ2, a genetic risk factor in AD, and reduces microglial phagocytosis and uptake of synaptic structures. As such BTK inhibition may represent a therapeutic route to prevent microglial activation and synapse loss in AD

Microglial-mediated synapse loss has been implicated in AD pathogenesis. Inhibition of BTK decreases activation of PLCγ2, a genetic risk factor in AD, and reduces microglial phagocytosis and uptake of synaptic structures. As such BTK inhibition may represent a therapeutic route to prevent microglial activation and synapse loss in AD

## Introduction

Central to the B cell receptor (BCR) signaling pathway, Bruton’s tyrosine kinase (BTK) is a cytoplasmic tyrosine kinase that has been well-characterized as a critical regulator of B cell development and activation (Jumaa et al. 2005). Loss-of-function mutations in *Btk* are responsible for X-linked agammaglobulinemia (XLA), a rare human primary immunodeficiency that results from incomplete B cell differentiation (Vetrie et al. 1993). Targeting BTK activity via small molecule covalent inhibitors like ibrutinib has also shown therapeutic efficacy in B cell malignancies associated with dysfunctional BCR signaling including mantle cell lymphoma (MCL) and chronic lymphocytic leukemia (CLL) (Advani et al. 2013; Hendriks et al. 2014). In addition to its role in adaptive immunity, BTK has increasingly been associated in multiple innate immune biologies. XLA patients and CLL patients receiving ibrutinib are at high risk of infection (Winkelstein et al. 2006; Williams et al. 2018) and that XLA patients can develop neutropenia due to impaired neutrophil maturation (Kozlowski and Evans 1991; Farrar et al. 1996). In mutant *Btk*-carrying X-linked immunodeficient (XID) mice, clearance of microfilaria by BTK-deficient macrophages is delayed (Mukhopadhyay et al. 2002) while in *Btk* knockout mice, macrophage/monocyte numbers are reduced (Melcher et al. 2008). Furthermore, BTK has been implicated in Toll-like receptor (TLR)-mediated proinflammatory cytokine release from macrophages and dendritic cells following lipopolysaccharide (LPS) challenge (Schmidt et al. 2006; Ni Gabhann et al. 2012; Ni Gabhann et al. 2014).

As the innate immune cells of the central nervous system (CNS), microglia are the resident phagocytes responsible for surveying their local environment and responding in case of CNS injury or pathogen entry (Salter and Beggs 2014). Ontogenically distinct from other mononuclear phagocytes, microglia originate early in development from erythromyeloid progenitor cells in the embryonic yolk sac that migrate into the brain before the blood-brain barrier (BBB) is formed (Ginhoux et al. 2010). Unlike their peripheral myeloid counterparts, CNS microglia are also long-living with a low homeostatic turnover during adulthood (Reu et al. 2017). Moreover microglia are unique immune cells in performing non-classical functions such as sculpting neuronal circuits during development by engulfing and removing excess synapses and neurons (Stevens et al. 2007; Wakselman et al. 2008). This mechanism relies on classic immune molecules such as complement proteins like complement receptor 3 (CR3) on microglia to phagocytose and eliminate immature synapses that have been tagged by C1q and C3 (Schafer et al. 2012). More recently evidence has emerged that microglial-mediated synaptic pruning pathways may be reactivated during disease. In mouse models of Alzheimer’s disease (AD) (Hong et al. 2016; Shi et al. 2017) and frontotemporal dementia (FTD) (Lui et al. 2016), elevated levels of complement factors cause early synaptic loss which can be rescued by inhibition or deletion of C1q, C3 or CR3. Interestingly, reduced CR1- and CR3-mediated phagocytosis has been reported in monocytes derived from XLA patients (Amoras et al. 2003). Microglia can also damage surrounding neurons by releasing proinflammatory molecules in response to build-up of protein aggregates or ongoing neuronal loss. Amyloid-β (Aβ)-induced activation of TLRs and the NLRP3 (NACHT, LRR, and PYD domain-containing protein 3) inflammasome results in production and release of proinflammatory cytokines like IL-1β and genetic deletion of NLRP3 protects against Aβ pathology and cognitive dysfunction in AD mouse models (Heneka et al. 2013). In this context BTK has also recently been implicated as a direct regulator of NLRP3 inflammasome activation and IL-1β release in murine macrophages and human peripheral blood mononuclear cells (PBMCs) (Ito et al. 2015; Liu et al. 2017).

Given its role in various myeloid cell functions and its promise as a drug target, understanding the expression and potential impact of BTK in microglia functions holds great interest. Here we investigate BTK expression in microglia in vitro and in vivo and use potent BTK inhibitors and BTK-targeting small interfering RNA (siRNA) to assess the contribution of BTK to various microglial phenotypes implicated in human disease including phagocytosis, synaptic uptake, migration, cytokine release and morphology. The role of BTK in neurodegenerative disease has not been well explored to date. Human genetic studies over the last decade have identified common and rare variants in genes highly expressed in microglia that contribute to AD risk such as *TREM2*, *CR1*, *SORL1*, *CD33* as well as *PLCG2*, whose protein product phospholipase gamma 2 (PLCγ2) is a BTK substrate (Watanabe et al. 2001; Bertram et al. 2008; Reitz et al. 2011; Sims et al. 2017). Here we assess BTK levels in two mouse models of AD and in two gene expression datasets from post-mortem AD patient brain tissues.

## Methods

### Compounds and siRNA

CC-292 (Selleckchem) and ibrutinib (Medchem Express) were used at indicated concentrations dissolved in 100% dimethyl sulfoxide (DMSO; Sigma-Aldrich, D8418). DMSO at 0.1% (*v/v*) was used as vehicle control in all the in vitro experiments. LPS from *Escherichia coli* (O55:B5) was obtained from Sigma-Aldrich and used at a concentration of 1 μg/ml for in vitro experiments. The siRNA pool for BTK knockdown was obtained from Dharmacon and Silencer® negative control No. 1 siRNA was obtained from ThermoFisher.

### Cell Culture

The BV2 mouse microglia cell line (Banca Biologica e Cell Factory, ICLC) was grown in complete medium: DMEM GlutaMAX (ThermoFisher) supplemented with 10% foetal bovine serum (FBS; ThermoFisher) and 1% penicillin/streptomycin (P/S; ThermoFisher) at 37 °C in 5% CO_2_ in a humidified incubator. For isolation of primary microglia, forebrains were first isolated from post-natal day 7–8 (P7–8) mice (C57BL/6J; Charles River) and meninges carefully removed. Brains were dissociated using the Papain Dissociation System (Worthington) according to the manufacturer’s instructions. Homogenates were filtered through a 40 μm cell strainer (Falcon) and resuspended in complete medium. Single cell suspensions were then transferred into T75 flasks and incubated at 37 °C in 5% CO_2_ for 7 days. Microglia were isolated from mixed glial cell cultures by shaking flasks for 1 h at 200 rpm at 37 °C, re-suspended in complete medium with 20 ng/ml carrier-free macrophage colony stimulating factor (M-CSF; ThermoFisher) and grown for 7 days in 2-well culture insert 24-well (Ibidi) or 96-well (Greiner) plates. This protocol typically generates microglia cultures with >95% purity as assessed by immunocytochemistry for Iba1 (microglia marker) and GFAP (astrocyte marker; data not shown). Human monocytes were obtained with informed consent from healthy donors from the University of Nebraska Medical Center and cultured for 7 days in the presence of 20 ng/ml M-CSF.

### Synaptosome Purification

Brains were dissected from 3-month old Sprague-Dawley rats (Charles River), placed in 10 volumes of ice cold HEPES-buffered sucrose (0.32 M sucrose, 4 mM HEPES pH 7.4) and homogenized using a Dounce homogenizer. Homogenate was spun at 1000 x g at 4 °C for 10 mins to remove the pelleted nuclear fraction (P1). The resulting supernatant was spun at 15,000 x g for 20 mins to yield a crude synaptosomal pellet (P2) which was resuspended in 10 volumes of HEPES-buffered sucrose. After centrifugation at 10,000 x g for an additional 15 mins, the washed crude synaptosomal fraction (P2’) was layered onto 4 ml of 1.2 M sucrose and centrifuged at 230,000 x g for 15 mins. The interphase was collected, layered onto 4 ml of 0.8 M sucrose and centrifuged at 230,000 x g (SW40 Ti rotor, Beckman Optima L-90 K) for 15 mins to yield the synaptosome pellet. Purified synaptosomes were conjugated with pH-sensitive rhodamine-based pHrodo® Red succinimidyl ester (ThermoFisher, P36600) in 0.1 M sodium carbonate (pH 9.0) by incubation for 2 h at room temperature with gentle agitation. Unbound pHrodo® was removed by multiple rounds of washing and centrifugation with HBSS and pHrodo®-conjugated synaptosomes were then resuspended in HBSS with 5% DMSO and stored at −80 °C until use.

### In Vitro Phagocytosis Assays

BV2 cells and primary microglia were plated at a density of 20,000 cells in 96-well (Greiner) plates. Cells were treated for 2 h or 24 h with 0.1- or 1 μM of CC-292 or ibrutinib (both in DMSO) and then incubated for 1 h with pHrodo®-conjugated zymosan bioparticles (12.5 μg/ml per well; ThermoFisher) or pHrodo®-labeled purified synaptosomes (5 μl). For siRNA treatment, BV2 microglia were transfected with 25 nM of negative control non-targeting (NT) or BTK siRNA using ViromerBlue (Lipocalyx) for 24 h followed by incubation for 1 h with zymosan particles. After washing cells with PBS, cells were fixed in 4% paraformaldehyde (PFA) for 10 mins at room temperature. Fixed cells were stained with either HCS CellMask Blue or Alexa Fluor 488 Phalloidin with DAPI (ThermoFisher) to enable accurate cell segmentation and zymosan particle counting. Images were acquired using the Leica TCS SP5 II confocal microscope and IN Cell Analyzer 6000 system (GE Healthcare Life Sciences) with cell segmentation and particle counting performed using the IN Cell Developer Toolbox v1.9. Phagocytic index was measured as outlined below:

$$ \mathrm{Phagocytic}\ \mathrm{Index}=\left(\mathrm{No}.\mathrm{of}\ \mathrm{particles}/\mathrm{cell}\right)\ \mathrm{x}\ \left(\%\mathrm{of}\ \mathrm{phagocytic}\ \mathrm{cells}\right) $$

For synaptosome uptake measurements in primary microglia, co-localization of pHrodo®-red signal and CellMask Blue was performed using a custom MATLAB application and results expressed using the Pearson coefficient.

### Scratch Wound Migration Assay

Primary microglia were seeded at a density of 30,000 cells/insert in 2-well culture insert 24-well plates (Ibidi). Cells were incubated at 37 °C in 5% CO_2_ until reaching approximately 80% confluence. Culture-inserts were then carefully removed followed by washing of the cell monolayer with fresh complete medium and imaging of the scratch area using an EVOS digital inverted light microscope. Primary microglia were treated with 1 μM of CC-292 for 24 h and the scratch area re-imaged. The extent of microglia cell migration into the scratch area was quantified using ImageJ.

### Enzyme-Linked Immunosorbent Assay

Supernatants from vehicle- or CC-292-treated primary microglia were measured for cytokine levels using the Meso Scale Discovery (MSD) V-Plex assay kit following manufacturer’s instructions.

### Western Blots

BV2 and mouse primary microglia were washed with ice-cold PBS and lysed in RIPA buffer (Sigma-Aldrich) containing protease and phosphatase inhibitors (Roche). Hemibrains from 6-month old 5xFAD mice (Oakley et al. 2006) and littermate controls were obtained from QPS (Austria) and lysed in RIPA buffer with inhibitors using a cordless pestle motor. Cortical brain tissue from 7-month old Thy1-hTau.P301S mice (Allen et al. 2002) and littermate controls was obtained from reMYND (Belgium) and lysed in RIPA buffer with inhibitors using a cordless pestle motor. Cell and tissue lysates were centrifuged at 10,000 x g for 20 mins at 4 °C and supernatants used for BCA measurements (Pierce BCA Protein Assay Kit) of protein concentration. Equal amounts of protein in Laemmli sample buffer (Biorad) were separated by gel electrophoresis using 4–12% Bis-Tris polyacrylamide gels (Invitrogen) and transferred to PVDF membranes (MerckMillipore). Nonspecific binding was blocked by incubating membranes for 1 h in TBS blocking buffer (Odyssey, Licor) followed by overnight incubation at 4 °C with the following primary antibodies: rabbit anti-phospho-BTK (pBTK; Abcam, ab68217), anti-BTK (CST, 8547S), anti-phospho-PLCγ2 (pPLCγ2; CST, 3871S), anti-PLCγ2 (CST, 3872S), anti-SV2A (Abcam, ab32942), anti-PSD-95 (Abcam, ab18258), anti-MBP (Merck Millipore, MAB386) and anti-β-actin (Licor). Blots were washed 3 times in 1X TBS with 0.1% Tween-20 (TBS-T) followed by incubation for 1 h with fluorescent secondary antibodies (Licor) at room temperature. After 3 additional washes in TBS-T, blots were imaged using the Odyssey CLx Imaging system (Licor). ImageJ was used to quantitate protein levels using corresponding actin levels as loading control.

### Ex Vivo Phagocytosis Assay Using Acute Mouse Brain Slices

C57BL/6J mice were anaesthetized with isoflurane, decapitated and brains carefully dissected. 300 μm-thick sagittal sections were cut using a vibratome (Leica VT 1200 S) in ice-cold carbogen (95% CO_2_, 5% O_2_)-bubbled artificial cerebrospinal fluid solution (aCSF) consisting of 126 mM choline chloride, 3 mM KCl, 2.4 mM CaCl_2_, 1.3 mM MgCl_2_, 26 mM NaHCO_3_, 1.24 mM NaH_2_PO_4_, and 10 mM glucose. Slices were immediately placed for 1 h at 35 °C in incubation chambers (Prechamber BSC-PC, Harvard Apparatus) filled with carbogen-bubbled aCSF (with choline chloride replaced by a stoichiometric amount of NaCl). Slices were then incubated with 1 μM of CC-292 for 2 h at 37 °C followed by incubation with pHrodo®-conjugated zymosan bioparticles (500 μg/ml, 100 μl per slice; ThermoFisher) at 37 °C for an additional hour. After washing slices with PBS, they were fixed in 4% PFA for 1 h at room temperature before proceeding to immunostaining. Fixed slices were incubated for 3 h with blocking solution (normal goat serum 5%, 0.05% Triton-X in PBS) and incubated for 48 h with anti-Iba1 (Synaptic Systems, 234,004; 1:500 dilution). Slices were washed three times for 15 mins in 1X PBS and incubated with secondary antibody (anti-guinea pig IgG, Alexa488; ThermoFisher) for 3 h at room temperature. After washing in 1X PBS, slices were counterstained with DAPI (ThermoFisher) and mounted. Images were acquired using the Zeiss LSM880 confocal microscope and the number of particles engulfed by Iba1-positive microglia counted.

### Immunohistochemistry and Immunocytochemistry

Brain cryosections from 6-month old 5xFAD mice and littermate controls were obtained from QPS (Austria). Briefly, isolated brains were immersion-fixed in 4% PFA for 2 h at room temperature followed by 15% sucrose in PBS overnight at 4 °C. Brains were embedded in OCT medium (Tissue-Tek) and snap-frozen in ice-cooled liquid isopentane. Sagittal cryosections were cut at 10 μm thickness on a Leica cryotome and processed for immunohistology. Non-specific binding was blocked with 10% normal donkey serum (ThermoFisher) in 0.3% Triton X-100/PBS for 1 h. Sections were incubated overnight at 4 °C with the following primary antibodies: anti-Iba1 (Synaptic Systems, 234,004; 1:500), anti-BTK (CST, 8547S; 1:100), and anti-Aβ 4G8 (Biolegend, 800,702; 1:500). Sections were then washed three times for 15 mins in 1X PBS followed by incubation with fluorophore-conjugated highly cross-absorbed secondary antibodies (Abcam, Jackson ImmunoResearch) for 1 h at room temperature. After washing in 1X PBS, sections were counterstained with DAPI. For immunocytochemistry, BV2 cells were fixed in 4% PFA for 10 mins at room temperature, blocked using 3% bovine serum albumin (BSA) and incubated overnight with anti-BTK (1:100). All images were obtained using the Leica TCS SP5 II confocal microscope using LAS X imaging software or the Zeiss LSM880 confocal microscope using Zeiss ZEN 2.3 imaging software. BTK/Iba1 co-localization was measured using the UCB custom application in MATLAB as outlined above.

### Microglia Morphology Analysis

Multiple images were captured from cortical, hippocampal and striatal regions of ex vivo acute mouse brain slices using the Zeiss Axioscan and processed using Zeiss ZEN 2.3 software. These images were then used for microglia morphology analysis using the ImageJ plugin *NeurphologyJ Interactive* (Ho et al. 2011) to measure microglial soma number and process number/length.

### Statistical Analysis

Results are presented as means ± standard error of the mean (s.e.m.). Statistics were calculated using GraphPad Prism 7. Data were analyzed using two-tailed Student’s *t* test to compare between two groups or one-way ANOVA with Tukey’s post-hoc multiple comparison test to compare between several groups.

## Results

### BTK Expression and Inhibition in Microglia In Vitro and In Vivo

**Fig. 1:**
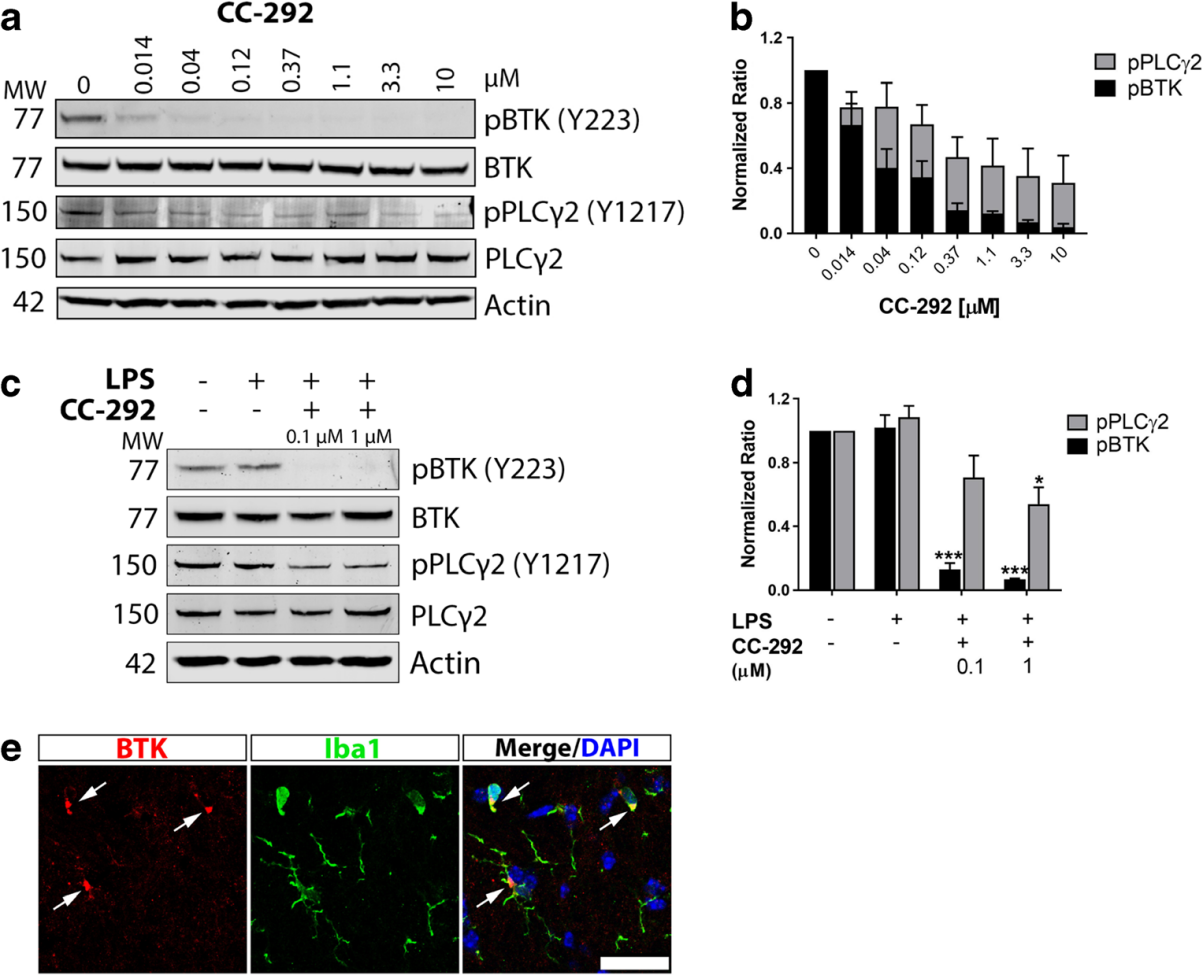
BTK expression and inhibition in microglia in vitro and in vivo. **a** Western blot of protein lysates from BV2 cells incubated with increasing concentrations of CC-292 for 2 h. CC-292 treatment demonstrated potent concentration-dependent silencing of BTK activity as measured by pBTK levels and reduced PLCγ2 activation as measured by pPLCγ2 levels. **b** Quantification of pBTK (normalized to total BTK) and pPLCγ2 (normalized to total PLCγ2) protein levels in BV2 cells incubated with increasing concentrations of CC-292. Protein levels quantified from 3 independent experiments. **c** Western blot of protein lysates from primary mouse microglia incubated with LPS and 0.1 or 1 μM of CC-292 for 24 h. CC-292 treatment inhibited BTK and reduced PLCγ2 activation. **d** Quantification of pBTK (normalized to total BTK) and pPLCγ2 (normalized to total PLCγ2) protein levels in primary mouse microglia incubated with LPS and 0.1 or 1 μM of CC-292. Protein levels quantified from 3 independent experiments. Data presented as mean ± s.e.m., one-way ANOVA with Tukey’s post-hoc multiple comparison test, *p < 0.05, ***p < 0.001 versus LPS vehicle control. **e** Representative confocal microscopy images of BTK immunoreactivity in Iba1-positive microglia in brains of wild-type mice. Scale bar = 25 μm

Previous RNA-sequencing analyses have indicated that BTK transcript is highly expressed in microglia relative to other cell types in both the mouse and human brain (Zhang et al. 2014, 2016). To assess BTK protein levels in microglia and potential for inhibition of the microglial BTK pathway, we treated both immortalized mouse microglia (BV2) and primary microglia with increasing concentrations of CC-292, a highly selective and potent inhibitor of BTK kinase activity (IC_50_ = 0.5 nM) (Evans et al. 2013). Detection of BTK by immunoblot using an anti-BTK antibody was confirmed in BV2 microglia and CC-292 treatment potently inhibited BTK autophosphorylation on Tyr223 in these cells (Fig. 1a, b). Furthermore downstream phosphorylation of PLCγ2, a BTK substrate (Watanabe et al. 2001), was lowered by CC-292 treatment (Fig. 1a, b). Similarly in primary microglia stimulated with LPS, CC-292 treatment inhibited BTK and reduced PLCγ2 activation (Fig. 1c, d). We then immunostained for BTK in wild-type C57BL/6 brains and found a clear overlap of BTK immunoreactivity with Iba1-positive cells (Fig. 1e).

### BTK Inhibition or Knockdown Decreases Phagocytosis in Rodent Microglia and Human Monocyte-Derived Macrophages

**Fig. 2:**
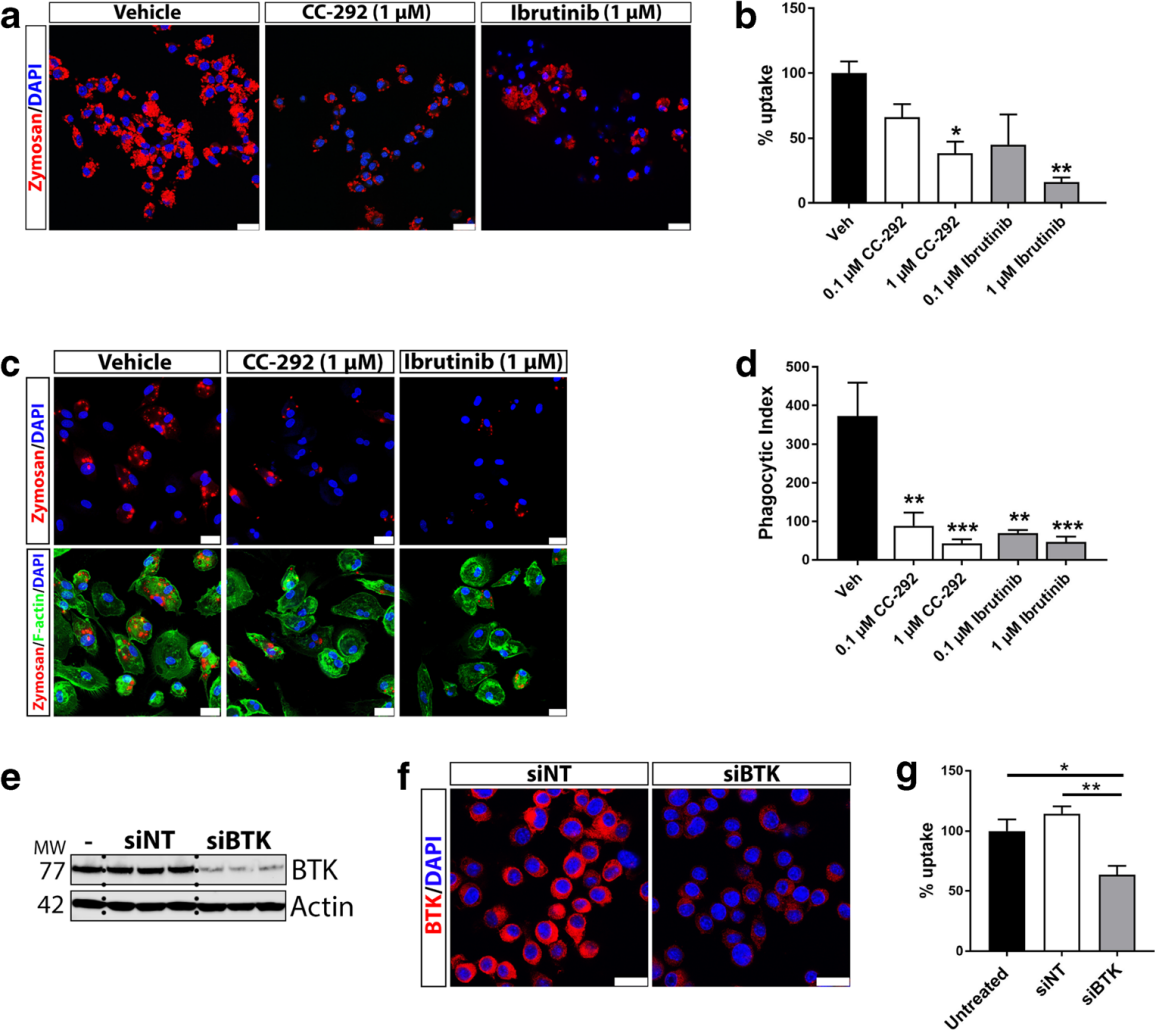
BTK inhibition or BTK knockdown decreases phagocytosis in BV2 microglia and human monocyte-derived macrophages. **a** Representative confocal microscopy images of BV2 cells incubated with 1 μM of CC-292 or ibrutinib for 2 h followed by incubation with pHrodo® zymosan particles for 1 h. Scale bar = 25 μm. **b** Uptake of zymosan particles was significantly reduced following treatment of BV2 cells with 1 μM of CC-292 or ibrutinib. **c** Representative confocal microscopy images of human monocyte-derived macrophages incubated with 1 μM of CC-292 or ibrutinib for 24 h followed by incubation with pHrodo® zymosan particles for 1 h. Scale bar = 25 μm. **d** Uptake of zymosan particles was significantly reduced following treatment of human monocyte-derived macrophages with CC-292 or ibrutinib. **e** Transfection of BV2 microglia with BTK siRNA potently downregulates BTK protein levels compared to non-targeting (NT) siRNA-transfected or untransfected cells as measured by Western blot. **f** Immunocytochemistry of BTK in BV2 cells transfected with NT or BTK siRNA. Scale bar = 25 μm. **g** BTK knockdown in BV2 microglia using BTK siRNA decreases phagocytosis of zymosan particles. Data presented as mean ± s.e.m., 4 biological replicates for each condition, *n* = 300–350 analyzed cells per condition, one-way ANOVA with Tukey’s post-hoc multiple comparison test, *p < 0.05, **p < 0.01 versus vehicle control

**Fig. 3:**
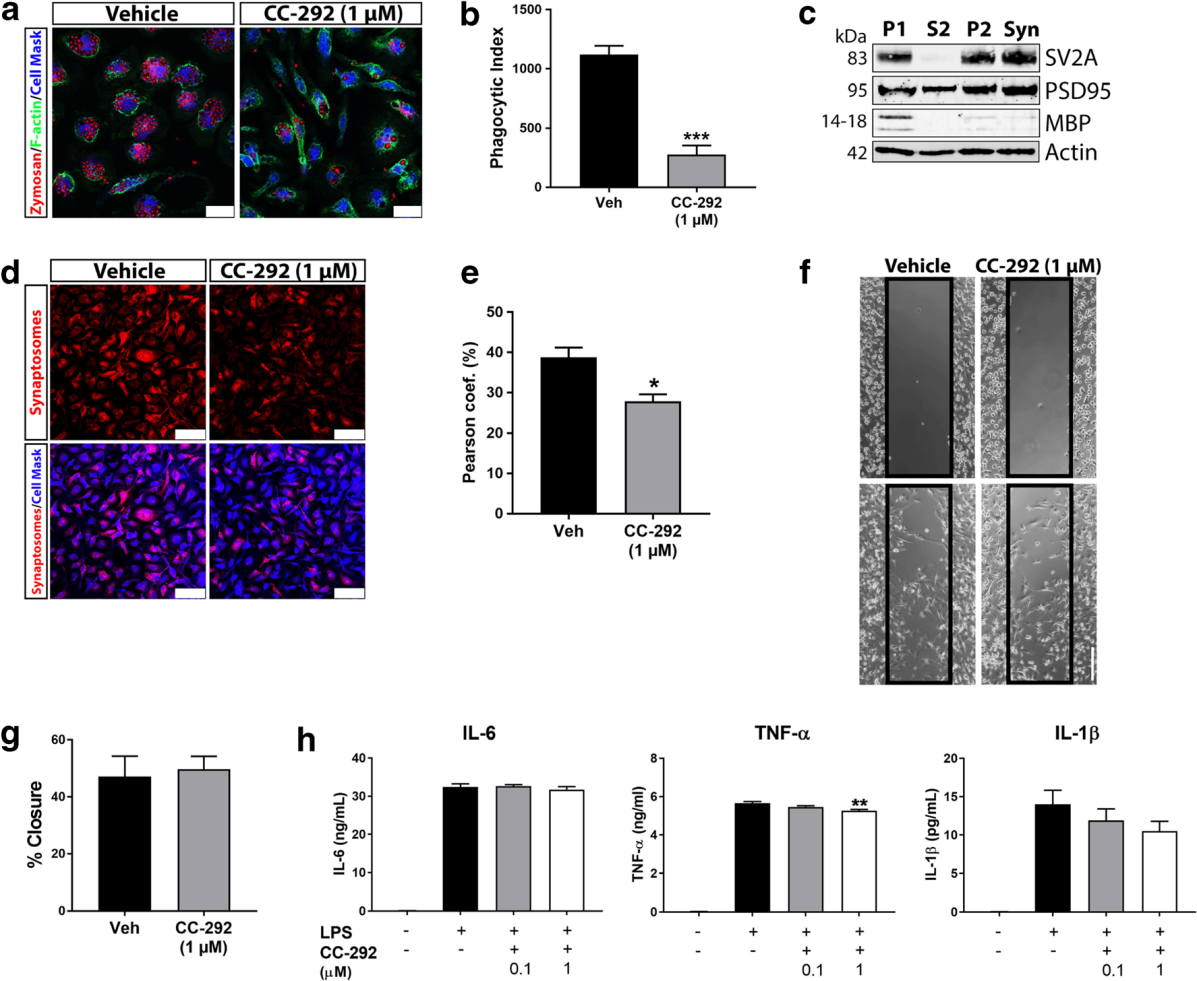
Inhibition of BTK reduces microglial uptake of synaptic structures with minimal impact on migration and cytokine release. **a** Representative confocal microscopy images of primary microglia incubated with 1 μM of CC-292 for 24 h followed by incubation with pHrodo® zymosan particles for 1 h. Cells were stained with phalloidin and CellMask to allow for cell segmentation. Scale bar = 25 μm. **b** Phagocytosis of zymosan particles was significantly reduced following CC-292 treatment as measured by the phagocytic index. **c** Western blot of rat brain synaptosome preparations showing enrichment of SV2A (presynaptic marker) and PSD-95 (postsynaptic marker) with low MBP (myelin) signal in the synaptosome (Syn) fraction. **d** Representative confocal microscopy images of primary microglia incubated with 1 μM of CC-292 for 24 h followed by incubation with pHrodo®-conjugated purified synaptosomes for 1 h. Cells were stained with CellMask to allow for cell segmentation. Scale bar = 75 μm. **e** Synaptosome uptake was significantly reduced following CC-292 treatment as measured by co-localization with CellMask Blue. **f** Representative light microscopy images from scratch migration assay of primary microglia incubated with 1 μM of CC-292 for 24 h. Scale bar = 200 μm. **g** Ability of microglia to migrate into scratch was not significantly different between vehicle and CC-292 treatment. **h** Cytokine release from LPS-stimulated primary microglia incubated with 0.1 or 1 μM of CC-292 for 24 h. IL-6 levels were unchanged between vehicle and CC-292 treatment in LPS-stimulated cells. A small but significant decrease in TNF-α was observed with 1 μM of CC-292. Non-significant decreases in IL-1β were measured with CC-292 treatment. Data presented as mean ± s.e.m., 4 biological replicates for each condition, *n* = 300–350 analyzed cells per condition for phagocytosis assays, unpaired *t*-test (phagocytosis and migration assays), one-way ANOVA with Tukey’s post-hoc multiple comparison test (cytokine release), *p < 0.05, **p < 0.01, ***p < 0.001 versus vehicle control (for cytokine analysis, versus LPS vehicle control)

Having detected BTK protein expression in microglia in vitro and in vivo, we next sought to determine the functional role(s) of BTK in microglia. Previous studies have indicated that BTK is involved in the phagocytosis of various pathogen substrates by macrophages (Byrne et al. 2013; Strijbis et al. 2013). To assess the ability of microglia to phagocytose after BTK inhibition, BV2 microglia were treated with CC-292 or a second BTK inhibitor ibrutinib (Advani et al. 2013) followed by incubation with pHrodo®-conjugated zymosan particles. Uptake of zymosan particles was significantly reduced following treatment of BV2 cells with 1 μM of CC-292 or ibrutinib (Fig. 2a, b). Similarly, in human monocyte-derived macrophages treated with CC-292 or ibrutinib, zymosan phagocytosis was significantly lower (Fig. 2c, d). To confirm that inhibition of BTK function modulates phagocytosis and to exclude the possibility of non-specific effects of BTK inhibitors, BV2 microglia were transfected with BTK-targeting siRNA which led to potent knockdown of BTK levels (Fig. 2e, f). BTK knockdown significantly decreased zymosan uptake in BV2 microglia when compared to NT siRNA- or untransfected cells (Fig. 2g). To assess whether BTK inhibition also impacts phagocytosis in primary microglia, isolated microglia from brains of post-natal mouse pups were treated with CC-292 (1 μM) followed by incubation with pHrodo®-conjugated zymosan particles. Quantification of zymosan uptake revealed a significant reduction in phagocytosis by primary microglia following CC-292 treatment (Fig. 3a, b). Thus, blockade of BTK function reduces phagocytosis in both rodent microglia and human macrophages.

### Inhibition of BTK Reduces Microglial Uptake of Synaptic Structures with Minimal Impact on Migration and Cytokine Release

Next, in order to understand BTK-associated phagocytosis in a CNS-relevant context, we isolated synaptosomes from rat brains by density gradient centrifugation. Western blot analyses verified the purity of synaptosome preparations showing enrichment of presynaptic (SV2A) and postsynaptic (PSD-95) markers with minimal myelin (MBP) content (Fig. 3c). Synaptosomes were then labeled with pHrodo® and applied to primary microglia cultures treated with CC-292 (1 μM). Co-localization analysis indicated a significant decrease in phagocytic uptake of synaptic particles in CC-292-treated cells (Fig. 3d, e). Since BTK and PLCγ2 are known to regulate B cell migration (de Gorter et al. 2007), we next sought to measure BTK involvement in microglia cell migration using a scratch invasion assay. We found that CC-292 treatment of primary microglia did not impact cell invasion of the scratch over a 24 h period (Fig. 3f, g). BTK has also been identified as a direct regulator of NLRP3 inflammasome activation and IL-1β release in murine macrophages and human PBMCs (Ito et al. 2015; Liu et al. 2017). Using ELISA we measured levels of IL-1β and other proinflammatory cytokines, IL-6 and TNF-α, in supernatants of LPS-stimulated microglia treated with CC-292. While IL-1β levels were dose-dependently lower with CC-292 treatment compared to LPS-stimulated vehicle, this result was not significant (Fig. 3h). IL-6 levels were unchanged between vehicle and CC-292 treatment in LPS-stimulated microglia while a small but significant decrease in TNF-α was observed with 1 μM of CC-292 (Fig. 3h). Since LPS stimulation did not significantly affect pBTK and pPLCγ2 levels (Fig. 1c, d), these results suggest that BTK does not play a major role in LPS-mediated cytokine release in microglia.

### BTK Inhibition Decreases Microglial Phagocytosis and Alters Microglial Morphology Ex Vivo

**Fig. 4:**
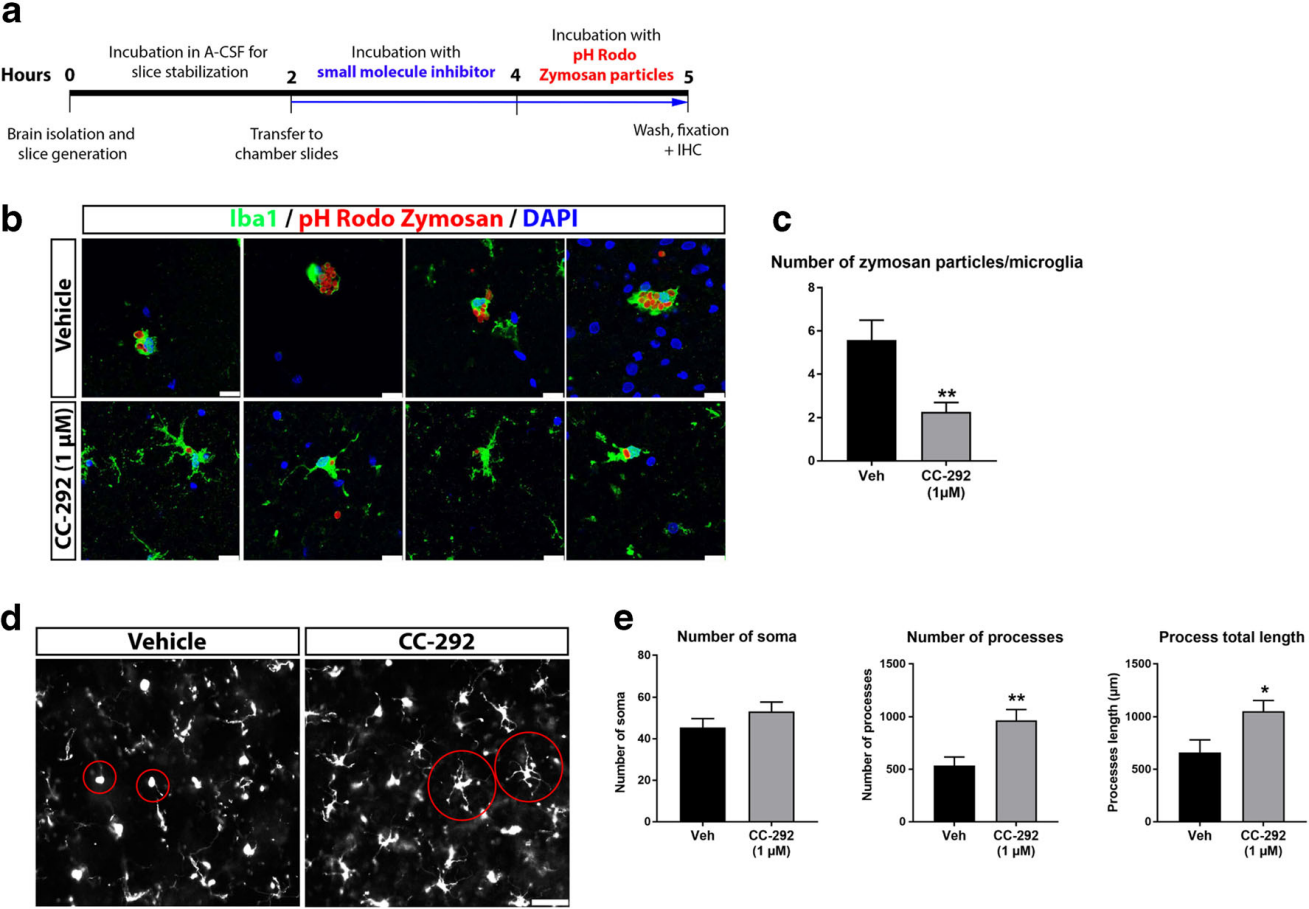
BTK inhibition decreases microglial phagocytosis and alters microglial morphology ex vivo. **a** Schematic diagram of the ex vivo microglial phagocytosis assay using acute rat brain slices. **b** Representative confocal images of acute rat brain slices incubated with 1 μM of CC-292 for 2 h followed by incubation with pHrodo® zymosan particles for 1 h. Uptake of zymosan particles by microglia was visualized by Iba1 immunostaining. Scale bar = 25 μm. **c** Number of zymosan particles phagocytosed per microglia was significantly reduced following CC-292 treatment. **d** Representative Axioscan images of Iba1-positive microglia in acute rat brain slices incubated with CC-292. Scale bar = 50 μm. **e** Measurements of microglia number and morphology showed significantly increased number of microglial processes and increased process length in CC-292-treated brain slices. Data presented as mean ± s.e.m., *n* = 5–6 slices per condition, unpaired *t*-test, *p < 0.05, **p < 0.01 versus vehicle control

Cellular assays of BTK function in microglia highlighted a predominant role for BTK in phagocytosis. To exclude the possibility that these effects were due to in vitro conditions, we next sought to investigate whether BTK also regulates microglial phagocytosis in the brain ex vivo*.* To examine this we adopted an acute brain slice method in order to explore microglial activation, phagocytosis and morphology in a more complex system that retains the brain cytoarchitecture (Fig. 4a). In line with our in vitro findings, we found that phagocytic activity in CC-292-treated acute rat brain slices was lower compared to vehicle treatment as measured by the number of zymosan particles per Iba1-positive cell (Fig. 4b, c). Differences in microglia morphology were also apparent in CC-292-treated brain slices (Fig. 4d). Resting microglia are generally characterized by a ramified cell morphology with long branching processes whereas phagocytic microglia hold an amoeboid shape (Boche et al. 2013). We quantified the number of microglia soma, process number and process length using *NeurphologyJ* software and found a significantly increased number of microglial processes and increased process length in CC-292-treated brain slices whereas microglial number was unaltered (Fig. 4e). This increase in microglial process number and length following CC-292 treatment potentially reflects the reduced rates of phagocytosis (and hence fewer amoeboid morphologies) measured after BTK inhibition.

### Elevated BTK Levels in Brains of 5xFAD Mice and AD Patients

**Fig. 5:**
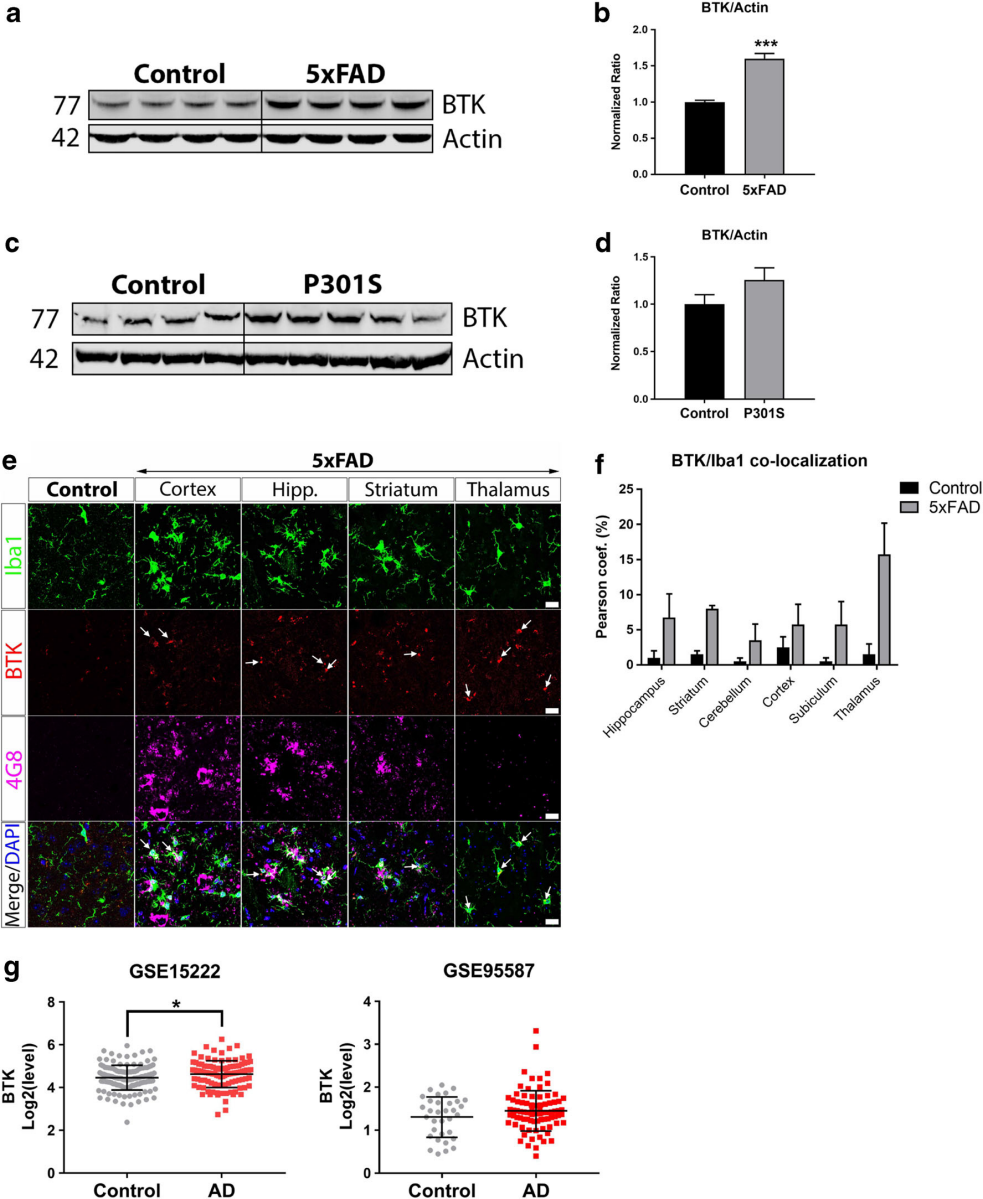
Elevated BTK levels in brains of 5xFAD mice and AD patients. **a** Western blot of protein lysates isolated from brains of 5xFAD mice and littermate controls. **b** Quantification and normalization of BTK levels to actin loading control shows significantly increased BTK in 5xFAD brains. Data presented as mean ± s.e.m., *n* = 4 animals per group, unpaired *t*-test, ***p < 0.001 versus littermate control. **c** Western blot of protein lysates isolated from cortical brain tissue of P301S mice and littermate controls. **d** Quantification and normalization of BTK levels to actin loading control shows a non-significant increase in BTK levels in P301S brains. Data presented as mean ± s.e.m., *n* = 4–5 animals per group, unpaired *t*-test. **e** Representative confocal microscopy images of BTK immunoreactivity in Iba1-positive microglia in control brains and in indicated regions of 5xFAD mouse brains showing Aβ (4G8) immunoreactivity. Scale bar = 20 μm. **f** BTK/Iba1 co-localization analysis showed increased microglial BTK levels in multiple brain regions of 5xFAD mice compared to littermate controls. Data presented as mean ± s.e.m., *n* = 4 animals per group. **g** BTK transcript levels in two previously published gene expression datasets from AD patient brain bulk tissues (GSE15222: temporal cortex, GSE95587: fusiform gyrus). BTK levels were modestly elevated in AD patients in both datasets with a significant increase of BTK in dataset GSE15222. Data presented as mean ± s.d., unpaired *t*-test, *p < 0.05

Having uncovered a role for BTK in activating microglial PLCγ2, an AD-associated risk factor (Sims et al. 2017), and in regulating microglial phagocytosis including the uptake of synaptic structures, we next wished to investigate whether BTK is involved in the pathophysiology of AD, a neurodegenerative disease in which microglia have been proposed to mediate synaptic loss (Hong and Stevens 2016). Using the 5xFAD transgenic model of severe amyloid pathology (Oakley et al. 2006), we examined the expression of BTK in brain tissues from 6-month old 5xFAD mice, an age at which synaptic loss and microglial activation is apparent (Oakley et al. 2006). Western blot analysis revealed that BTK levels were significantly elevated in the 5xFAD brains compared to littermate controls (Fig. 5a, b). Next, we analyzed BTK levels in the Thy1-hTau.P301S tauopathy mouse model (Allen et al. 2002). In 7-month old P301S transgenic mice, an age in which there is cortical neurodegeneration and extensive neuroinflammation (Bellucci et al. 2004; Hampton et al. 2010), BTK was upregulated though this result was not statistically significant (Fig. 5c, d). Localization of the BTK signal by immunohistochemistry confirmed increased BTK immunoreactivity in Iba1-positive microglia in 5xFAD brain sections compared to littermate controls (Fig. 5e, f). Finally we used the recently created Brain Myeloid Landscape platform (Friedman et al. 2018) to assess BTK transcript levels in two separate gene expression datasets from AD patient bulk brain tissues: GSE15222 in temporal cortex measured by microarray (Webster et al. 2009) and GSE95587 in fusiform gyrus measured by RNA-sequencing (Friedman et al. 2018) (Fig. 5g). In both datasets, BTK levels were elevated in AD patient brains relative to age-matched controls with a significant elevation in dataset GSE152222.

## Discussion

In recent years the role of innate immunity in AD pathogenesis has received considerable attention based on diverse evidence from human genetic studies (Huang et al. 2017; Sims et al. 2017), microglia RNA-seq transcriptional profiling (Keren-Shaul et al. 2017; Yin et al. 2017) and studies depleting microglia in AD mouse models (Olmos-Alonso et al. 2016; Spangenberg et al. 2016). These combined findings have revealed a complex temporal and functional involvement of microglia in AD risk and progression: while neuroprotective roles of microglia in limiting Aβ build-up may act in presymptomatic AD (Wang et al. 2015; Zhao et al. 2018), once in motion the disease course may be accelerated by microglial-mediated spreading of tau pathology and synapse loss (Asai et al. 2015; Hong et al. 2016; Hansen et al. 2018). Phagocytosis is a central microglial function across these different disease stages and therefore identifying microglia/infiltrating myeloid drug targets that impact phagocytic uptake holds great potential in AD treatment. In our current study we have found that inhibition of BTK modulates microglial phagocytosis in vitro and ex vivo with downstream effects on microglial morphology. Furthermore BTK is upregulated in the 5xFAD mouse model and in post-mortem AD patient brains. BTK elevation in the 5xFAD mouse model and human AD brains may therefore represent a neuroinflammatory response to extracellular Aβ accumulation, synaptic loss or multiple pathologies. Interestingly, a recent whole exome sequencing study of AD patients has identified a protective genetic variant in a downstream substrate of BTK, *PLCG2* (P522R) (Watanabe et al. 2001; Sims et al. 2017). Whether this variant represents a gain- or loss-of-function mechanism is currently under investigation. Some preliminary work indicates that the functional impact of the P522R variant may block the active site of PLCγ2 (Menzies et al. 2017), however another report points to a small hypermorphic effect of this variant on enzymatic activity with no obvious impact on PLCγ2 expression relative to the wild-type allele (Magno et al. 2018). Understanding the impact of *PLCG2* coding variants will shed further light on the potential of targeting BTK pathways in AD.

Here we have found that BTK regulates the phagocytic uptake of zymosan particles by rodent microglia and human monocyte-derived macrophages in vitro and in ex vivo acute brain slices. TLR2 and Dectin-1, both upregulated in human AD tissues (Webster et al. 2009; Friedman et al. 2018) and AD mouse models (Wes et al. 2014; Holtman et al. 2015), have been recognized as pattern recognition receptors for zymosan and separate reports have implicated BTK as an essential downstream component of both TLR2 and Dectin-1 signaling pathways in macrophages (Horwood et al. 2006; Strijbis et al. 2013). Along other phagocytic pathways, BTK mediates clearance of Aspergillus infection as part of a TLR9-calcineurin-NFAT axis (Herbst et al. 2015) while data exists both supporting (Amoras et al. 2003; Jongstra-Bilen et al. 2008; Mirsafian et al. 2017) and opposing (Ren et al. 2016; Cavaliere et al. 2017) a role for BTK in FcγR-mediated phagocytosis in monocytes and macrophages. In our present study inhibition of microglial BTK also reduced synaptosome uptake, however the precise synaptic components and signaling pathways that allow microglia to recognize and engulf specific synapses are not well understood (Hong and Stevens 2016). Significant work in recent years has highlighted that tagging of synapses with C1q or C3 promotes their removal by microglia via a CR3-dependent mechanism (Hong et al. 2016; Shi et al. 2017). While we did not coat synaptosome fractions with C1q or C3 in this study, a recent report has shown that C1q is present in synaptosomes prepared from wild-type mouse brains using a similar sucrose density centrifugation method (Gyorffy et al. 2018). Intriguingly, Byrne and colleagues (Byrne et al. 2013) also found that CD91-mediated uptake of C1q-opsonised apoptotic cells depends on BTK phosphorylation of calreticulin. In BTK-deficient macrophages, calreticulin fails to localize with CD91 at the phagocytic cup and uptake of C1q-opsonised apoptotic cells is impaired (Byrne et al. 2013). Upstream of BTK, Fc receptors are known to interact with complement factors such as C5a (Schmidt and Gessner 2005; Karsten and Kohl 2012) and reduced Fc-, CR1- and CR3-mediated phagocytosis has also been reported in monocytes derived from XLA patients (Amoras et al. 2003). Since complement receptor expression is normal or even elevated in monocytes from XLA patients (Amoras et al. 2007), this indicates that impaired phagocytosis in XLA patients may be due to altered cytoplasmic transduction mechanisms, though whether BTK is directly responsible is currently unknown. Nevertheless the potential involvement of BTK in complement-mediated synaptic loss is intriguing and warrants further attention. Excessive microglial cytokine production is another innate immune phenotype that may exacerbate AD pathology (Heneka et al. 2013; Maphis et al. 2015). Previous preclinical work in ischaemic stroke has found that the BTK inhibitor ibrutinib inhibits IL-1β maturation and NLRP3 inflammasome activation in infiltrating macrophages and neutrophils in the infarct area of the MCAO model (Ito et al. 2015). Furthermore in bone marrow-derived macrophages it has been shown that BTK is involved in LPS-mediated cytokine release (Schmidt et al. 2006; Ni Gabhann et al. 2014). Here we found that inhibition of BTK had a modest impact on the release of IL-1β from LPS-stimulated microglia. Similar to previous findings in LPS-stimulated PBMCs and macrophages from XLA patients (Horwood et al. 2003, 2006), we find that BTK may play a role in TLR4-induced TNF-α but not IL-6 production. However in this current study, since LPS stimulation did not significantly affect microglial pBTK and pPLCγ2 levels and BTK inhibition only modestly reduced TNF-α levels, these results indicate BTK may not play a major role in LPS-mediated cytokine release in microglia.

One caveat to the approach of targeting BTK in AD is the potentially protective role of B cells and adaptive immunity in modulating microglial response and limiting Aβ pathology (Marsh et al. 2016). As a result, CNS indications such as brain disease in systemic lupus erythematosus (SLE) or multiple sclerosis where both macrophage and B cell biologies are implicated in disease pathogenesis may be alternative options for BTK inhibition. In MRL/lpr mice that exhibit systemic autoimmune disease similar to human SLE, treatment with a BTK inhibitor ameliorated cognitive function and reduced accumulation of T cells, B cells and macrophages in the CNS (Chalmers et al. 2018). To date, peripheral chronic inflammatory diseases and blood cancers have been the primary clinical applications of BTK inhibitors currently approved or in development (Campbell et al. 2018). There are however studies in patients with primary CNS lymphoma (Lionakis et al. 2017), MCL (Bernard et al. 2015) and CCL (Wanquet et al. 2016) with CNS metastases, and Bing Neel syndrome (Mason et al. 2017) which indicate that ibrutinib may show BBB penetration and clinical efficacy in the CNS. The most-advanced BTK inhibitors in the clinic including ibrutinib are covalent and irreversible binders at a cysteine residue (Cys-481) in the ATP-binding pocket of BTK (Crawford et al. 2018). Since 10 other human kinases contain the same cysteine residue in their active site, off-target inhibition with covalent BTK inhibitors could present an issue in treating chronic neurodegenerative and neuroinflammatory disorders that require good safety profiles (Crawford et al. 2018). As such targeting BTK with potent, specific, reversible and brain-penetrant small molecules may allow for pharmacological inhibition of detrimental microglial functions in CNS disease.

In conclusion we have identified a role for BTK in regulating microglial phagocytosis. More research is needed to better define the contribution of BTK and other microglial targets to the phagocytosis of specific substrates in vivo particularly in AD mouse models that display synapse loss. Collectively, our study suggests that the development of brain penetrant small molecule BTK inhibitors may offer a therapeutic option in treating AD and other neurodegenerative and neuroinflammatory diseases associated with microglial activation and synaptic loss.
